# Eilat virus (EILV) causes superinfection exclusion against West Nile virus (WNV) in a strain-specific manner in Culex tarsalis mosquitoes

**DOI:** 10.1099/jgv.0.002017

**Published:** 2024-08-27

**Authors:** Renuka E. Joseph, Jovana Bozic, Kristine L. Werling, Rachel S. Krizek, Nadya Urakova, Jason L. Rasgon

**Affiliations:** 1 Department of Entomology, Pennsylvania State University, University Park, PA, USA; 2 Center for Infectious Disease Dynamics, Pennsylvania State University, University Park, PA, USA; 3 The Huck Institutes of the Life Sciences, Pennsylvania State University, University Park, PA, USA; 4 Department of Biochemistry and Molecular Biology, Pennsylvania State University, University Park, PA, USA

**Keywords:** alphavirus, *Culex tarsalis*, Eilat virus, flavivirus, heterologous interference, insect-specific virus, superinfection exclusion, West Nile virus

## Abstract

West Nile virus (WNV) is the leading cause of mosquito-borne illness in the USA. There are currently no human vaccines or therapies available for WNV, and vector control is the primary strategy used to control WNV transmission. The WNV vector *Culex tarsalis* is also a competent host for the insect-specific virus (ISV) Eilat virus (EILV). ISVs such as EILV can interact with and cause superinfection exclusion (SIE) against human pathogenic viruses in their shared mosquito host, altering vector competence for these pathogenic viruses. The ability to cause SIE and their host restriction make ISVs a potentially safe tool to target mosquito-borne pathogenic viruses. In the present study, we tested whether EILV causes SIE against WNV in mosquito C6/36 cells and *C. tarsalis* mosquitoes. The titres of both WNV strains – WN02-1956 and NY99 – were suppressed by EILV in C6/36 cells as early as 48–72 h post-superinfection at both m.o.i. values tested in our study. The titres of WN02-1956 at both m.o.i. values remained suppressed in C6/36 cells, whereas those of NY99 showed some recovery towards the final timepoint. The mechanism of SIE remains unknown, but EILV was found to interfere with NY99 attachment in C6/36 cells, potentially contributing to the suppression of NY99 titres. However, EILV had no effect on the attachment of WN02-1956 or internalization of either WNV strain under superinfection conditions. In *C. tarsalis*, EILV did not affect the infection rate of either WNV strain at either timepoint. However, in mosquitoes*,* EILV enhanced NY99 infection titres at 3 days post-superinfection, but this effect disappeared at 7 days post-superinfection. In contrast, WN02-1956 infection titres were suppressed by EILV at 7 days post-superinfection. The dissemination and transmission of both WNV strains were not affected by superinfection with EILV at either timepoint. Overall, EILV caused SIE against both WNV strains in C6/36 cells; however, in *C. tarsalis*, SIE caused by EILV was strain specific potentially owing to differences in the rate of depletion of shared resources by the individual WNV strains.

## Author Summary

West Nile virus (WNV) is the main cause of mosquito-borne disease in the USA. In the absence of a human vaccine or WNV-specific antivirals, vector control is the key strategy to reduce WNV prevalence and transmission. The WNV mosquito vector, *Culex tarsalis*, is a competent host for the insect-specific virus Eilat virus (EILV). EILV and WNV potentially interact within the mosquito host, and EILV can be used as a safe tool to target WNV in mosquitoes. Here, we characterize the ability of EILV to cause superinfection exclusion (SIE) against two strains of WNV – WN02-1956 and NY99 – in C6/36 cells and *C. tarsalis* mosquitoes. EILV suppressed both superinfecting WNV strains in C6/36 cells. However, in mosquitoes, EILV enhanced NY99 whole-body titres at 3 days post-superinfection and suppressed WN02-1956 whole-body titres at 7 days post-superinfection. Vector competence measures, including infection, dissemination, and transmission rates and transmission efficacy, as well as leg and saliva titres of both superinfecting WNV strains, were not affected by EILV at both timepoints. Our data show the importance of not only validating SIE in mosquito vectors but also testing multiple strains of viruses to determine the safety of this strategy as a control tool.

## Introduction

West Nile virus (WNV) is a single-stranded, positive-sense RNA virus belonging to the genus *Orthoflavivirus* (family *Flaviviridae*) [1]; since its first report in the USA in New York City in 1999 [2,3], WNV has expanded its geographical prevalence throughout North America [4] and is now the predominant cause of mosquito-borne disease in the USA [5]. WNV is maintained in nature by an enzootic cycle between *Culex* mosquitoes and birds; however, spillovers into dead-end hosts such as humans and horses frequently occur, triggering epidemics [1]. The severity of symptoms in humans varies from asymptomatic WNV infection to neuroinvasive disease and death [5,6]. Since 1999, the USA has had 50 000 confirmed WNV cases, 25 000 neuroinvasive cases and 2000 deaths [5,6].

Since the first WNV outbreak in the USA in 1999, WNV has evolved, with the causative WNV genotype NY99 being displaced by the genotype WN02, which currently circulates in the US population [7,8]. The WN02 genotype sequence differs from the NY99 genotype sequence by three nucleotides [7,8]. Two of these mutations were in the envelope protein and one in the NS5 protein, but only one of these mutations was non-synonymous [7,8]. The WN02 genotype is characterized by a single amino acid change in the envelope protein from valine to alanine at position 159 (VE159A). This mutation, VE159A, enhances the vector competence of the WN02 genotype in *Culex* mosquitoes, particularly in *Culex tarsalis* [9] – the main WNV vector in rural areas [10]. *Culex pipiens* and *C. quinquefasciatus* are also key WNV vectors but mainly in urban settings [11]. Currently, no human vaccines or WNV-specific antivirals are available, making vector control the primary strategy used to reduce WNV transmission [12].

The WNV vector *C. tarsalis* is also a competent host for Eilat virus (EILV) [13], an insect-specific virus (ISV) belonging to the genus *Alphavirus* (family *Togaviridae*) [13]. EILV is a small, enveloped, single-stranded positive-sense RNA virus that is unable to infect vertebrate cells at both the attachment/entry and replication stages of the viral life cycle [14,15]. ISVs, including EILV, interact and modulate the vector competence of human pathogenic viruses in their shared mosquito hosts [16,22]. A deeper understanding of these interactions can potentially be used to develop these ISVs into safe tools to target pathogenic viruses in mosquitoes.

One such interaction of interest is superinfection exclusion (SIE), a phenomenon that occurs when a pre-existing viral infection in cells blocks or interferes with a secondary infection of the same virus (homologous interference) [23,25] or closely related virus [26,28], or even an unrelated virus (heterologous interference) [21,29, 30]. The precise mechanism(s) of SIE remains unknown. However, there is evidence suggesting that the primary virus impacts different stages of the virus life cycle of the challenge virus in cells, including attachment [31,32], penetration [24] and replication [33,34].

ISVs cause both homologous and heterologous interference in cell culture and in mosquitoes, although the results have been variable [19,20, 22, 33, 35,37]. For example, Palm Creek virus, an insect-specific flavivirus, when superinfected with WNV in C6/36 cells, caused heterologous interference against WNV [17] but Culex flavivirus Izabal, another insect-specific flavivirus, had no effect on WNV under similar conditions [38]. Previous studies have demonstrated that EILV causes homologous and heterologous interference against other alphaviruses in C7/10 cells and *Aedes aegypti* mosquitoes [22]. The superinfection of EILV-infected *A. aegypti* with Chikungunya virus (CHIKV) delayed CHIKV dissemination by 3 days [22]. The mosquito species *C. tarsalis* is a more competent host for EILV than *A. aegypti* [13] and therefore we proposed to investigate the ability of EILV to cause heterologous interference against the unrelated flavivirus WNV in C6/36 cells and *C. tarsalis* mosquitoes.

## Methods

### Cells and cell culture

The WNV- and EILV-susceptible *Aedes albopictus* mosquito cell line C6/36 was propagated in Roswell Park Memorial Institute (RPMI) medium, consisting of RPMI 1640 medium (Gibco/Thermo Fisher Scientific), supplemented with 10% (v/v) FBS (Gibco/Thermo Fisher Scientific), penicillin (100 U ml^−1^; Gibco/Thermo Fisher Scientific) and streptomycin (100 µg ml^−1^; Gibco/Thermo Fisher Scientific), and maintained at 28 °C with no CO_2_.

### Viral cDNA clone and virus propagation

#### Eilat virus

The EILV cDNA clone was obtained from the World Reference Center for Emerging Viruses and Arboviruses at the University of Texas Medical Branch (Galveston, TX, USA) and used for all experiments. The EILV cDNA clone consists of EILV strain EO329 with an eGFP inserted into the nsp3 hypervariable region of the viral genome (EILV-eGFP). The EILV-eGFP virus was rescued as previously described [13].

#### West Nile virus

WNV strain WN02-1956 (GenBank: AY590222) stocks were originally obtained from Dr Gregory Ebel at the Center for Vector-Borne Infectious Diseases, Colorado State University (Fort Collins, CO, USA), whereas WNV strain NY99 (GenBank: AF196835.2) stocks were acquired from Dr Laura Kramer at the Wadsworth Center, New York State Department of Health (Albany, NY, USA). WNV strains were amplified in C6/36 cells, and the virus-containing supernatant was collected at 7 days post-infection (dpi). The collected supernatant was aliquoted and stored at −80 °C until use. A focus-forming assay (FFA) was used to quantify both EILV and WNV titres (described below).

### Superinfection fluorescence imaging

#### EILV-WNV superinfection in C6/36 cells

C6/36 cells were seeded in two T75 tissue culture flasks (Corning/Falcon) at a density of 6×10^7^ cells and incubated overnight without CO_2_ at 28 °C until they reached ~70–80% confluency. Both T75 flasks were then washed with serum-free RPMI medium. One of the flasks was then inoculated with EILV-eGFP at an m.o.i. of 10, whereas the other flask was mock inoculated with serum-free RPMI medium; the flasks were incubated at 28 °C with no CO_2_ for 1 h. After incubation, the remaining inoculum in the flasks was removed and replaced with complete RPMI, and the flasks were incubated at 28 °C with no CO_2_ for 48 h.

The EILV-eGFP and mock-infected C6/36 cells were detached using trypsin (Gibco/Thermo Fisher Scientific) after the 48 h incubation and seeded in a 24-well tissue culture plate (Greiner Bio-One) at a density of 5×10^6^ cells in triplicate and incubated overnight at 28 °C with no CO_2_. The cells in both groups were washed with serum-free RPMI medium, infected with the WNV strain WN02-1956 or NY99 at an m.o.i. of 0.1 or 0.01, and incubated at 28 °C with no CO_2_ for 1 h. The cells were washed twice with complete RPMI medium after WNV infection to remove any unattached virus. Complete RPMI medium was then added to the cells, and the EILV–WN02-1956-superinfected or EILV–NY99-superinfected and the control WN02-1956-infected or NY99-infected cells at both m.o.i. values were incubated at 28 °C with no CO_2_ for 72 h.

Superinfected cells were fixed 72 h post-superinfection using 300 µl of 4% formaldehyde in 1× PBS for 30 min at room temperature (RT). Superinfected cells were washed twice with 500 µl of 1× PBS and then permeabilized by adding 300 µl of 0.2% triton-X in 1× PBS to each well for 15 min at RT. Then, the cells were washed twice with 1× PBS and blocked with 300 µl of 3% BSA in 1× PBS for 1 h at RT. The cells were washed again with 1× PBS twice; next, 250 µl of the primary antibody, monoclonal anti-flavivirus group antigen antibody (clone D1-4G2-4-15, diluted 1 : 500 in 3% BSA), was added to each well, and the cells were incubated overnight at 4 °C. Superinfected cells were then washed twice with 1× PBS, and 250 µl of the secondary antibody, goat anti-mouse IgG (H+L) highly cross-adsorbed secondary antibody (Alexa Fluor Plus 594, diluted 1 : 1000 in 3% BSA), was added to each well. The plates were wrapped in aluminium foil and incubated overnight at 4 °C. Finally, cells were washed twice and maintained in 300 µl of 1× PBS to prevent drying. EILV infection was observed under the FITC filter and WN02-1956 and NY99 infections under the TRITC filter on the ECHO Revolve microscope. FITC and TRITC images were merged (all scale bars correspond to 180 µm).

### *In vitro* superinfection assay

EILV-eGFP-infected C6/36 cells were superinfected with WN02-1956 or NY99 at m.o.i. values of 0.1 and 0.01 in 24-well plates as described above. The superinfection assay was carried out with technical triplicates of superinfected and control groups at both m.o.i. values. Superinfected and control WN02-1956-infected or NY99-infected cells at both m.o.i. values were maintained at 28 °C with no CO_2_ for 96 h. At 12, 24, 48, 72 and 96 h post-superinfection, 300 µl of supernatant was collected from each superinfection and control well and replaced with fresh medium. Samples were stored at −80 °C until use, and WNV titres in the samples were quantified using FFA (described below). The *in vitro* superinfection assay was performed in duplicate biological replicates using aliquots of the same EILV-eGFP and WNV stocks that underwent the same number of freeze–thaw cycles.

### Viral attachment and internalization assay

The viral attachment and internalization assay was performed as previously described with some modifications [25]. For the viral attachment assay, a 24-well tissue culture plate with EILV-eGFP-infected (m.o.i. 10) and mock-infected C6/36 cells was seeded as described above in duplicate and superinfected with WWN02-1956 or NY99 at an m.o.i. of 0.1 as described above. Superinfected cells were then incubated at 4 °C for 1 h to allow virus particles to attach but not enter the cells. Then, superinfected cells were washed with 1× PBS (Gibco/Thermo Fisher Scientific) and then washed twice with complete RPMI medium to remove any unattached WNV. Both groups of superinfected cells, with and without EILV-eGFP infection, were resuspended in 300 µl of TRIzol for RNA extraction.

Similarly, for the viral internalization assay, a 24-well plate with EILV-eGFP-infected and mock-infected C6/36 cells superinfected with WN02-1956 or NY99 at an m.o.i. of 0.1 was incubated at 28 °C with no CO_2_ for 1.5 h to allow attachment and internalization of WNV. Superinfected cells were then washed with 1× PBS followed by a high-salt buffer containing 50 mM Na_2_CO_3_ (pH 9.5; Fisher Chemicals, Thermo Fisher Scientific) and 1 M NaCl (Fisher Chemicals, Thermo Fisher Scientific) for 3 min at RT. The high-salt buffer was removed, and the superinfected cells were resuspended in 300 µl of TRIzol for RNA extraction. RNA was extracted from all samples using the phenol/chloroform extraction method as previously described [39] and DNA contamination in the extracted RNA was removed from the samples using the TURBO DNA-free kit (Invitrogen, Thermo Fisher Scientific). Total RNA was quantified using a NanoDrop ND-1000 (NanoDrop Technologies/Thermo Fisher Scientific), and WNV RNA in 60 ng of total RNA from each sample was quantified by reverse transcription-quantitative PCR (RT-qPCR; described below). The virus attachment and internalization assays were performed in duplicate biological replicates using aliquots of the same EILV-eGFP and WNV stocks that underwent the same number of freeze–thaw cycles.

### RT-qPCR

WNV RNA was quantified using the Qiagen rotor gene Q-compatible qScript One-Step SYBR Green RT-qPCR kit (Quantabio). The RT-qPCR was set up as per the manufacturer’s instructions, using WNV envelope protein-specific primers 5′-TTGCAAAGTTCCTATCTCGTCAG-3′ and 5′-ACATGCCTCCGAACAGTGAG-3′ for both reverse transcription and amplification of the target WNV sequence. The WNV polyprotein DNA fragment (1917–2338 bp) synthesized by IDT Technologies served as standards for the RT-qPCR, making a standard curve from 10^6^ to 10^3^ viral copies µl^–1^. All samples and standards were run in duplicate, and WNV titres were determined using the standard curve.

### Mosquitoes and mosquito rearing

EILV-eGFP and WNV-competent mosquitoes, *C. tarsalis* (KNWR strain), were used in all experiments in our study. The KNWR strain was obtained from Dr Christopher Barker at the University of California–Davis School of Veterinary Medicine (Davis, CA, USA). Uninfected mosquitoes were reared and maintained at the Millennium Sciences Complex (The Pennsylvania State University, University Park, PA, USA), whereas WNV-infected mosquitoes were maintained at the Eva J. Pell Biosafety Level 3 (BSL3) laboratory (The Pennsylvania State University), as previously described [13]. *C. tarsalis* KNWR were reared in 30×30×30 cm cages, and growth conditions for both KNWR adults and larvae were 25±1 °C, 16 : 8 h light/dark diurnal cycle, and 80% relative humidity. Larvae were fed TetraMin (Tetra), and all adult mosquitoes were fed with 10% sucrose solution-soaked cotton balls *ad libitum*.

### Mosquito superinfection assay

Adult *C. tarsalis* KNWR female mosquitoes (3–5 days post-emergence) were sugar-starved for 24 h and then fed an infectious blood meal consisting of 1 : 1 anonymous human blood (BioIVT) and 10^7^ f.f.u. ml^–1^ of EILV-eGFP (EILV-eGFP-infected group) or RPMI medium (mock-infected group) at 37 °C using a water-jacketed membrane feeder. Previous studies have shown that an EILV-eGFP dose of 10^7^ f.f.u. ml^–1^ leads to a robust EILV infection in ~70% of the blood-fed *C. tarsalis* KNWR mosquitoes [13]. After the infectious bloodmeal, mosquitoes from both groups were cold-anaesthetized, and fully engorged female mosquitoes were counted and sorted into different cardboard cup cages with 10% sucrose solution-soaked cotton balls until further experimentation. At 5 dpi, mosquitoes in both groups were allowed to lay eggs in plastic cups filled with deionized water and the eggs were then discarded. Both EILV-eGFP-infected and mock-infected mosquitoes were moved to the Eva J. Pell BSL3 laboratory at 6 dpi and sugar-starved for 24 h. Mosquitoes in both groups were then fed again at 7 dpi with an infectious blood meal containing 1 : 1 anonymous human blood and WN02-1956 or NY99 at a dose of 10^7^ f.f.u. ml^–1^. Fully engorged females from the superinfected and single-infected control groups were cold-anaesthetized and placed into cardboard cup cages with 10% sucrose solution-soaked cotton balls. These cages with WNV-infected mosquitoes were then double caged and maintained at 25±1 °C, 16 : 8 light/dark diurnal cycle and 80% relative humidity until assay timepoints at 3 and 7 days post-superinfection.

Whole bodies from both superinfected and singly infected control groups were collected 3 days post-superinfection and placed into 2 ml microcentrifuge tubes containing 300 µl mosquito diluent [20% FBS, 100 µg ml^−1^ streptomycin, 100 µg ml^−1^ penicillin, 50 µg ml^−1^ gentamicin (Gibco/Thermo Fisher Scientific), and 2.5 µg ml^−1^ amphotericin B (Gibco/Thermo Fisher Scientific) mixed in 1× PBS]. Samples were then homogenized using a battery-operated tissue homogenizer (VWR) and disposable pestles (Fisher scientific/Thermo Fisher Scientific). Similarly, at 7 days post-superinfection, mosquitoes from both groups were forced to salivate for 30 min at RT into a capillary glass tube containing a mixture of 1 : 1 FBS and 50% sucrose. After salivation, the saliva was expelled into a 2 ml microcentrifuge tube containing 100 µl mosquito diluent. Legs and bodies of superinfected and control mosquitoes were then collected in 2 ml microcentrifuge tubes containing 300 µl mosquito diluent. The legs and bodies were homogenized using the battery-operated homogenizer and disposable pestles, and all samples were stored at −80 °C until further processing. The WNV titres of all samples collected were quantified using FFA (described below). The mosquito superinfection assay was performed in duplicate biological replicates, and aliquots of the same EILV-eGFP and WNV stocks that had undergone the same number of freeze–thaw cycles were used in both replicates.

Using the FFA results, the following vector competence parameters were determined. The infection rate (IR) was calculated as the proportion of WNV-infected mosquitoes among the total number of EILV-eGFP or mock-infected mosquitoes; the dissemination rate (DIR), as the proportion of WNV-infected mosquitoes with WNV-positive legs; the transmission rate (TR), as the proportion of mosquitoes with WNV-positive saliva from those with WNV-positive legs; and the transmission efficacy (TE), as the proportion of mosquitoes with WNV-positive saliva among the total number of EILV-eGFP or mock-infected mosquitoes.

### Focus-forming assay

We quantified EILV-eGFP titres using FFA as previously described [13]. Similarly, WNV titres were quantified by FFA as previously described but with some modifications [21]. Plates (96-well) were seeded with C6/36 cells at a density of 1×10^5^ cells per well and incubated at 28 °C with no CO_2_ overnight. The complete RPMI medium from the wells was then removed. Samples from the *in vitro* and *in vivo* superinfection assays were then serially diluted in serum-free RPMI medium from 10^0^ to 10^−7^ and 10^0^ to 10^−3^, respectively, and 30 µl of each diluted sample was added to the prepared C6/36 cells in duplicate. Mosquito saliva samples were not diluted, and 30 µl of each sample was added directly to the prepared cells. The cells were then incubated at 28 °C with no CO_2_ for 1 h, after which the samples were removed, and the cells were covered with 100 µl of RPMI containing 0.8% methylcellulose (Sigma-Aldrich). The WNV-infected cells were incubated at 28 °C without CO_2_ for 48 h. At 48 h post-infection, the infected C6/36 cells were fixed using 50 µl of 4% formaldehyde (Sigma-Aldrich) in 1× PBS for 30 min at RT. Cells were washed twice with 100 µl 1× PBS and were then permeabilized with 30 µl of 0.2% triton-X in 1× PBS for 15 min at RT. The washing step was repeated, and the cells were blocked with 30 µl of 3% BSA in 1× PBS for 1 h at RT. The cells were washed again, and 30 µl of the primary antibody, monoclonal anti-flavivirus group antigen antibody [clone D1-4G2-4-15 (BEI resources), diluted 1 : 500 in 3% BSA], was added to each well. The cells were then incubated overnight at 4 °C. Cells were then washed to remove any unattached primary antibody, and 30 µl of the secondary antibody goat anti-mouse IgG (H+L) highly cross-adsorbed secondary antibody (Alexa Fluor Plus 594 diluted 1 : 1000 in 3% BSA) was added to each well. The plates were wrapped in aluminium foil and incubated overnight at 4 °C. After a final washing step, the cells were maintained in 100 µl of 1× PBS to prevent drying. Fluorescent foci of EILV-eGFP were counted using the FITC filter on the ECHO Revolve microscope, whereas WN02-1956 or NY99 foci were counted using the TRITC filter.

### Statistical analysis

A two-way ANOVA followed by the Tukey test was used to determine the difference between WNV replication kinetics under the superinfection and single infection conditions in C6/36 cells. The WNV viral titres from the post-attachment or internalization assay were compared using Mann–Whitney U tests. The WNV viral titres of the body, leg and saliva samples from superinfected and singly infected *C. tarsalis* were also compared using Mann–Whitney U tests. The differences in the WNV IR, DIR, TR, and TE post-superinfection in EILV-exposed or mock-exposed *C. tarsalis* mosquitoes were evaluated using Fisher’s exact test. GraphPad Prism version 9.0.4 was used to perform all statistical tests.

## Results

### EILV caused heterologous interference against WNV strains WN02-1956 and NY99 *in vitro*

To investigate the ability of EILV to cause SIE against WNV in cell culture, C6/36 cells were mock infected or infected with EILV-eGFP at an m.o.i. of 10. After EILV infection was established at 72 h post-infection, cells were superinfected with the WNV strain WN02-1956 or NY99 at m.o.i. values of 0.1 and 0.01. Superinfected cells at both m.o.i. values were also observed using fluorescence microscopy at 72 h post-superinfection to determine whether EILV-eGFP-infected cells were resistant to WNV infection. Moreover, differences in WN02-1956 or NY99 replication kinetics under superinfection and single infection conditions at 12, 24, 48, 72 and 96 h post-superinfection were determined to detect SIE in C6/36 cells.

**Fig. 1.**
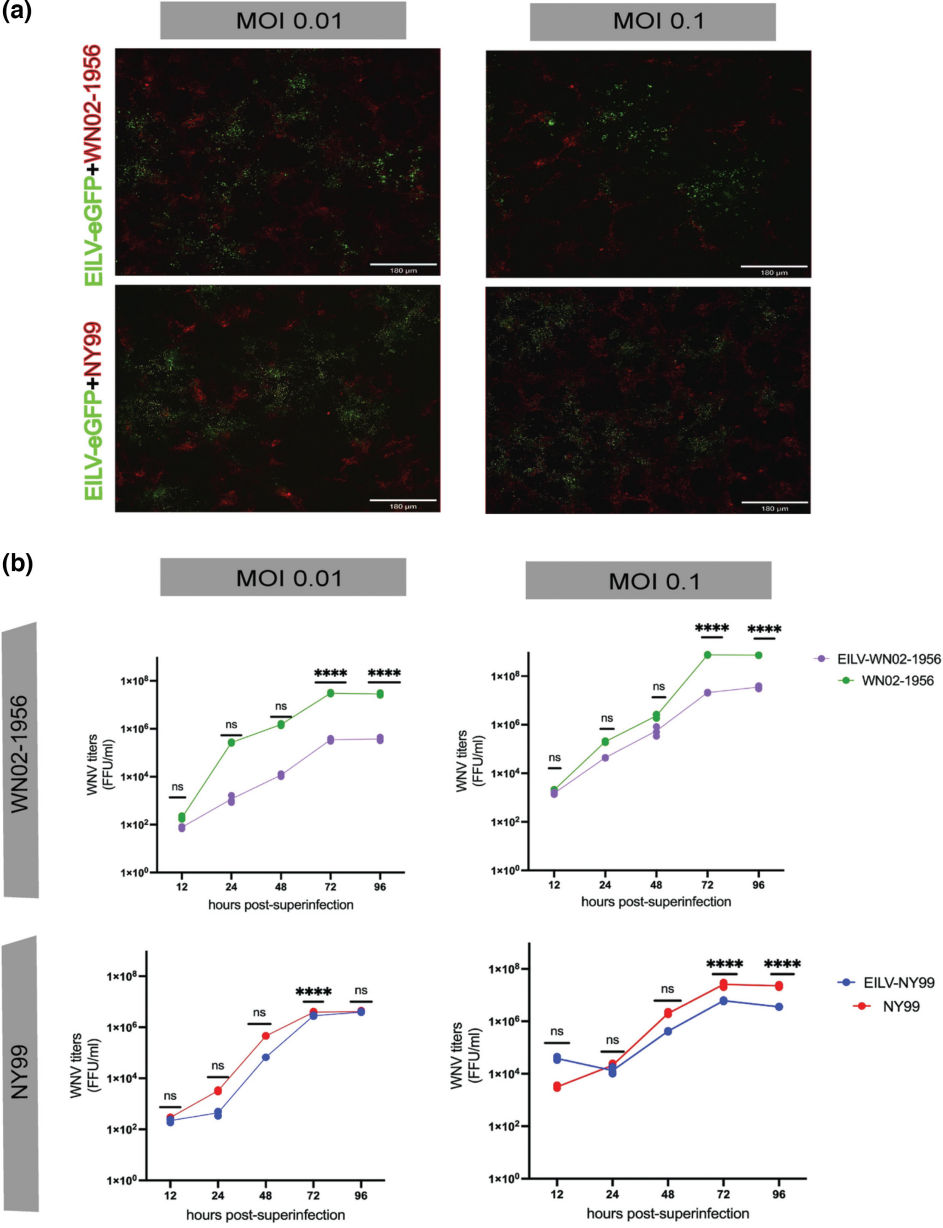
Superinfection exclusion (SIE) against WNV strains WN02-1956 and NY99 in C6/36 cells by EILV. (**a**) Superinfection of EILV-eGFP-infected C6/36 cells (m.o.i. of 10) with WN02-1956 or NY99 at an m.o.i. of 0.01 and 0.1 was visualized by fluorescence microscopy at 72 h post-superinfection. Representative images show eGFP (green) fluorescence for EILV-eGFP and Alexa Fluor 594 (red) fluorescence for both WNV strains. Bars, 180 µm. (**b**) Titres of WNV at 12, 24, 48, 72 and 96 h post-superinfection in EILV-eGFP-infected C6/36 cells (m.o.i. 10) and mock-infected cells superinfected with WN02-1956 or NY99 at an m.o.i. of 0.01 and 0.1 were determined by focus-forming assay (FFA). Each time point reflects the mean of technical triplicate infections. Statistical significance was evaluated using two-way ANOVA followed by Tukey test. **P<0.01 and ****P<0.0001.

We observed that C6/36 cells infected with EILV-eGFP, determined by eGFP expression by fluorescence microscopy, were susceptible to superinfection by WN02-1956 or NY99 virus, as indicated by Alexa Fluor 594 fluorescence at both m.o.i. values tested (Fig. 1a). The viruses also co-localized in the cells, suggesting co-infection by EILV and WN02-1956 or NY99 (Figs 1a and Fig. S1 [closeup image], available in the online version of this article).

We also found that the titres of WN02-1956 were statistically significantly lower at 72 and 96 h post-superinfection in EILV-eGFP-infected C6/36 cells than in mock-infected cells at both m.o.i. values tested (Fig. 1b, two-way ANOVA with Tukey test, all *P*<0.0001). A similar trend was observed in the second biological replicate of this assay, but with a significant suppression of WN02-1956 titres occurring earlier at 48 h in superinfected cells which continued at 72 and 96 h post-superinfection at both an m.o.i. of 0.1 (Fig. S2A; all *P*<0.0001) and 0.01 (Fig. S2A; *P*<0.0001, *P*<0.0001 and *P*≤0.01 respectively).

In contrast, the titre of the WNV strain NY99 was significantly lower in superinfected cells at an m.o.i. of 0.01 than in singly infected control cells only at 72 h post-superinfection (Fig. 1b; *P*<0.0001), with the NY99 titres rebounding at 96 h post-superinfection (Fig. 1b, *P*>0.05); however, the effect size was small and this might not be biologically significant. The second biological replicate of the EILV–NY99 superinfection assay at m.o.i. of 0.01 in C6/36 cells differed from the first replicate, with significantly lower NY99 titres observed at 48, 72 and 96 h post-superinfection (Fig. S2A; all *P*<0.0001). NY99 titres showed some but not significant recovery at 96 h post-superinfection, unlike that observed in the first replicate. Similarly, superinfection of EILV-eGFP-infected C6/36 cells with NY99 at m.o.i. of 0.1 led to significantly lower NY99 titres at 72 and 96 h post-superinfection (Fig. S2A, all *P*<0.0001) in superinfected cells than in singly infected control cells in both replicates. Overall, the WN02-1956 and NY99 titre reduction by EILV in C6/36 cells under superinfection conditions ranged between 10- and 100-fold.

### EILV interfered with the attachment of the superinfecting virus NY99 in C6/36 cells

Having established that EILV causes SIE against WN02-1956 and NY99 in C6/36 cells, we next investigated how EILV causes heterologous interference against unrelated WNV in cells. To this end, we assessed whether EILV interferes with the attachment or internalization stage of the WN02-1956 or NY99 viral life cycle in C6/36 cells. EILV-eGFP-infected and mock-infected C6/36 cells were superinfected with WN02-1956 or NY99 at an m.o.i. of 0.1. WNV particles were either allowed to only attach to their receptors or also internalize into the cells. WN02-1956 and NY99 RNA from superinfected and singly infected cells post-attachment or internalization were quantified by RT-qPCR to determine whether EILV interferes with either of these stages of the WNV life cycle.

**Fig. 2.**
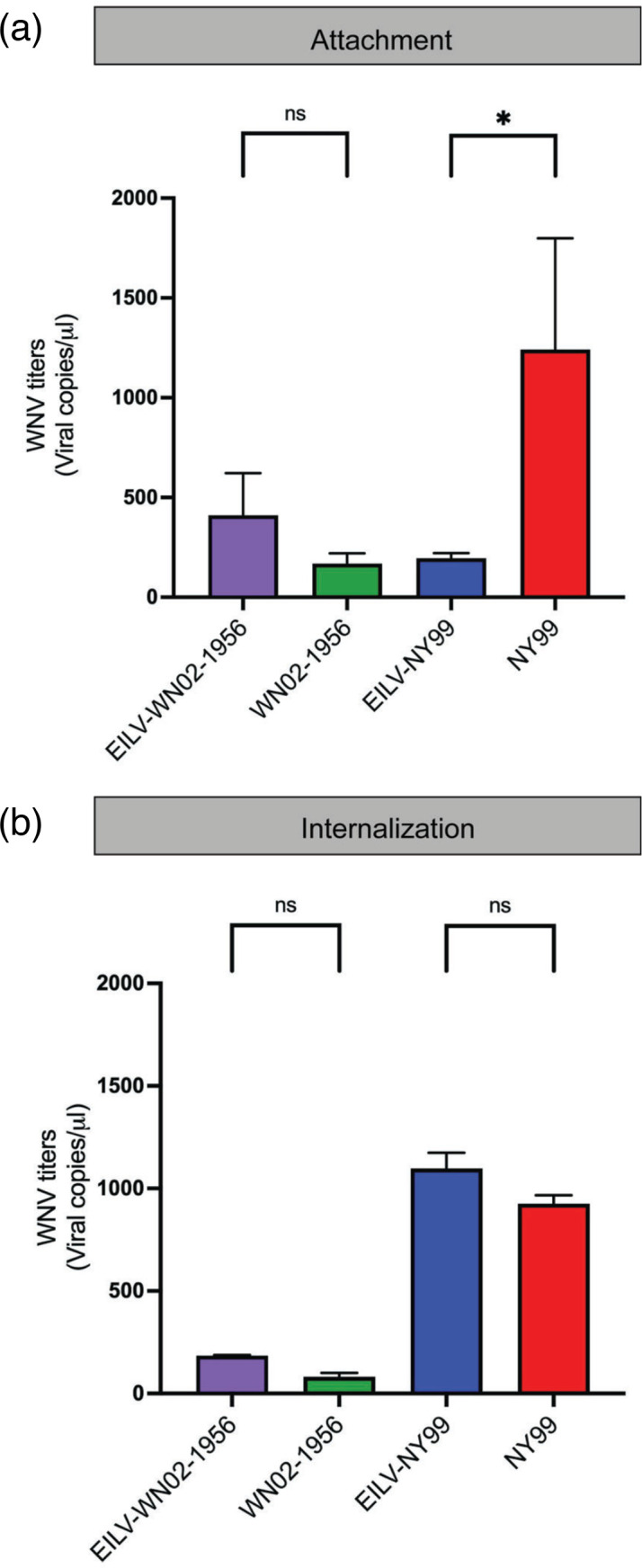
WNV attachment and internalization post-superinfection in EILV-eGFP-infected C6/36 cells. WNV levels (viral genome copies µl^–1^ extracted RNA) at (**a**) attachment and (**b**) internalization stages of the WNV life cycle in EILV-eGFP infected and mock-infected C6/36 cells superinfected with WN02-1956 or NY99 (m.o.i. 0.1) were determined by RT-qPCR. Each vertical bar reflects the mean of duplicate infections, and error bars indicate the standard deviation between replicates. Statistical significance was assessed using Mann–Whitney U tests. *P<0.05. Each point represents the mean of three replicates.

The viral levels of attached or internalized WN02-1956 in EILV-eGFP-infected C6/36 cells and in mock-infected C6/36 cells were not significantly different (Fig. 2a and b; respectively, Mann–Whitney U tests, both *P*>0.05). However, viral levels of attached NY99 were significantly lower in superinfected C6/36 cells than in singly infected cells (Fig. 2a; *P*<0.05). This difference between NY99 viral levels was not observed when comparing internalized virus particles in superinfected and singly infected cells (Fig. 2b; *P*>0.05).

### EILV enhanced NY99 whole-body titres at an early timepoint post-superinfection in *C. tarsalis* mosquitoes

We tested whether EILV causes heterologous interference against WNV strains in mosquitoes, as previously observed in C6/36 cells, by orally superinfecting EILV-eGFP-infected *C. tarsalis* KNWR mosquitoes with WN02-1956 or NY99. We examined the presence of EILV and WN02-1956 or NY99 using FFA in the bodies of 34 and 22 EILV-eGFP-challenged KNWR mosquitoes orally challenged with 10^7^ f.f.u. ml^–1^ of WN02-1956 or NY99, respectively, at 3 days post-superinfection. Simultaneously, for our controls, we examined the presence of WN02-1956 or NY99 using FFA at 3 days post-superinfection in the bodies of 29 and 19 mock-infected KNWR mosquitoes orally challenged with 10^7^ f.f.u.ml^–1^ of WN02-1956 or NY99, respectively. The IR for mosquitoes in our superinfected or mock-infected control groups was defined as the proportion of mosquitoes with WNV infection among the total number of EILV-exposed or mock-exposed mosquitoes or fully engorged females, respectively.

**Fig. 3.**
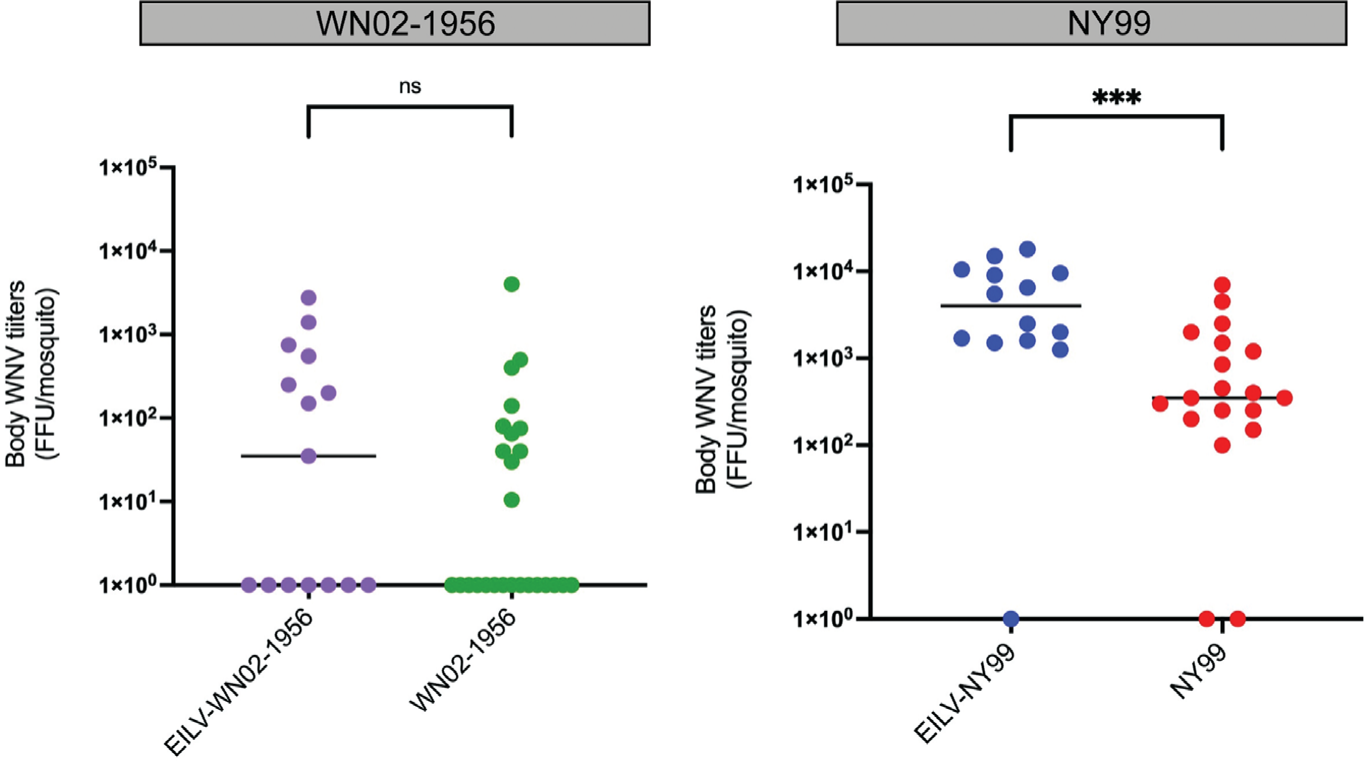
Whole-body WNV titres of superinfected and singly infected *Culex tarsalis* mosquitoes at 3 days post-superinfection. WNV viral titres of whole-body samples from EILV-eGFP-infected (10^7^ f.f.u. ml^–1^) and mock infected *C. tarsalis* mosquitoes orally challenged with (a) WN02-1956 and (**b**) NY99 (both 10^7^ f.f.u. ml^–1^) are plotted 3 days post-superinfection. Individual points represent a single mosquito sample, while group medians are depicted by horizontal bars. Significance was determined using Mann–Whitney U tests. ***P<0.001.

We observed that EILV-exposed *C. tarsalis* were not refractory to a secondary WN02-1956 or NY99 infection at 3 days post-superinfection (Table 1). At this early timepoint, the IR of WN02-1956- and NY99-superinfected mosquitoes did not differ significantly from the IR of mosquitoes singly infected with WN02-1956 or NY99 (Table 1; Fisher’s exact tests, both *P*>0.05). However, NY99 whole-body titres were significantly higher in EILV-exposed mosquitoes than in mock-exposed mosquitoes at 3 dpi (Fig. 3b; Mann–Whitney U tests, *P*≤0.001). No significant difference in WN02-1956 whole-body titres was observed at this timepoint between the two groups (Fig. 3a, *P*>0.05).

**Table 1.** Infection rate (IR) of Eilat virus (EILV)-eGFP-infected or mock-infected *Culex tarsalis* mosquitoes orally challenged with the West Nile virus (WNV) strain WN02-1956 or NY99 3 days post-superinfection

Superinfected mosquito group	IR (%) (*n*_I_/*n*_T_)
EILV-eGFP//WN02-1956	53.3 (8/15)
Mock-infected/WN02-1956	50 (11/22)
EILV-eGFP//NY99	92.8 (13/14)
Mock-infected/NY99	89.5 (17/19)

*n*_I_, number of mosquitoes infected with WNV; *n*_T_, total number of EILV-eGFP-positive or mock-infected mosquitoes tested.

### EILV suppressed WN02-1956 body titres at 7 days post-superinfection in *C. tarsalis* mosquitoes

We assessed the vector competence of WNV strains in EILV-exposed and mock-exposed *C. tarsalis* KNWR mosquitoes at a later timepoint to determine whether the ability of EILV to enhance or cause SIE changed with increasing time post-superinfection. We determined the IR, DIR, TR and TE of WN02-1956 in 37 EILV-exposed and 36 mock-exposed mosquitoes and of NY99 in 14 EILV-exposed and 24 mock-exposed mosquitoes at 7 days post-superinfection. IR was calculated as previously defined; DIR was determined as the proportion of EILV- or mock-infected mosquitoes with WNV-positive legs, TR as the proportion of EILV- or mock-infected mosquitoes with WNV-positive legs that also have WNV-positive saliva, and TE as the proportion of EILV- or mock-infected mosquitoes with WNV-positive saliva.

We found that the IR and DIR of WN02-1956 and NY99 in superinfected mosquitoes did not differ significantly from mock-infected mosquitoes at this timepoint (Table 2; Fisher’s exact test, *P*>0.05). Both WNV strains disseminated past the midgut in EILV- and mock-infected mosquitoes (Table 2, *P*>0.05), but WN02-1956 and NY99 were detected only in the saliva of a single mock-infected mosquito. However, the difference in the TR and TE of WN02-1956 and NY99 in EILV- and mock-infected mosquitoes was not statistically significant (Table 2, *P*>0.05).

**Table 2.** Infection rate (IR), dissemination rate (DIR), and transmission rate (TR) and transmission efficiency (TE) of Eilat virus (EILV)-eGFP-positive or mock-infected *C. tarsalis* mosquito species orally challenged with the West Nile virus (WNV) strain WN02-1956 or NY99 7 days post-superinfection

Superinfected mosquito group	IR (%) (*n*_I_/*n*_T_)	DIR (% (*n*_L_/*n*_I_)	TR (%) (*n*_S_/*n*_L_)	TE (%) (*n*_S_/*n*_T_)
EILV-eGFP/WN02-1956	84.2 (16/19)	25 (4/16)	0 (0/4)	0 (0/19)
Mock-infected–WN02-1956	87.5 (28/32)	28.6 (8/28)	12.5 (1/8)	3.1 (1/32)
EILV-eGFP/NY99	100 (14/14)	28.6 (4/14)	0 (0/4)	0 (0/14)
Mock-infected–NY99	100 (24/24)	50 (12/24)	8.3 (1/12)	4.2 (1/24)

*n*_I_, number of mosquitoes infected with WNV; *n*_T_, total number of EILV–eGFP-positive or mock-infected mosquitoes tested; *n*_L_, number of mosquitoes with WNV-positive legs; *n*_s_, number of mosquitoes with WNV-positive saliva.

**Fig. 4.**
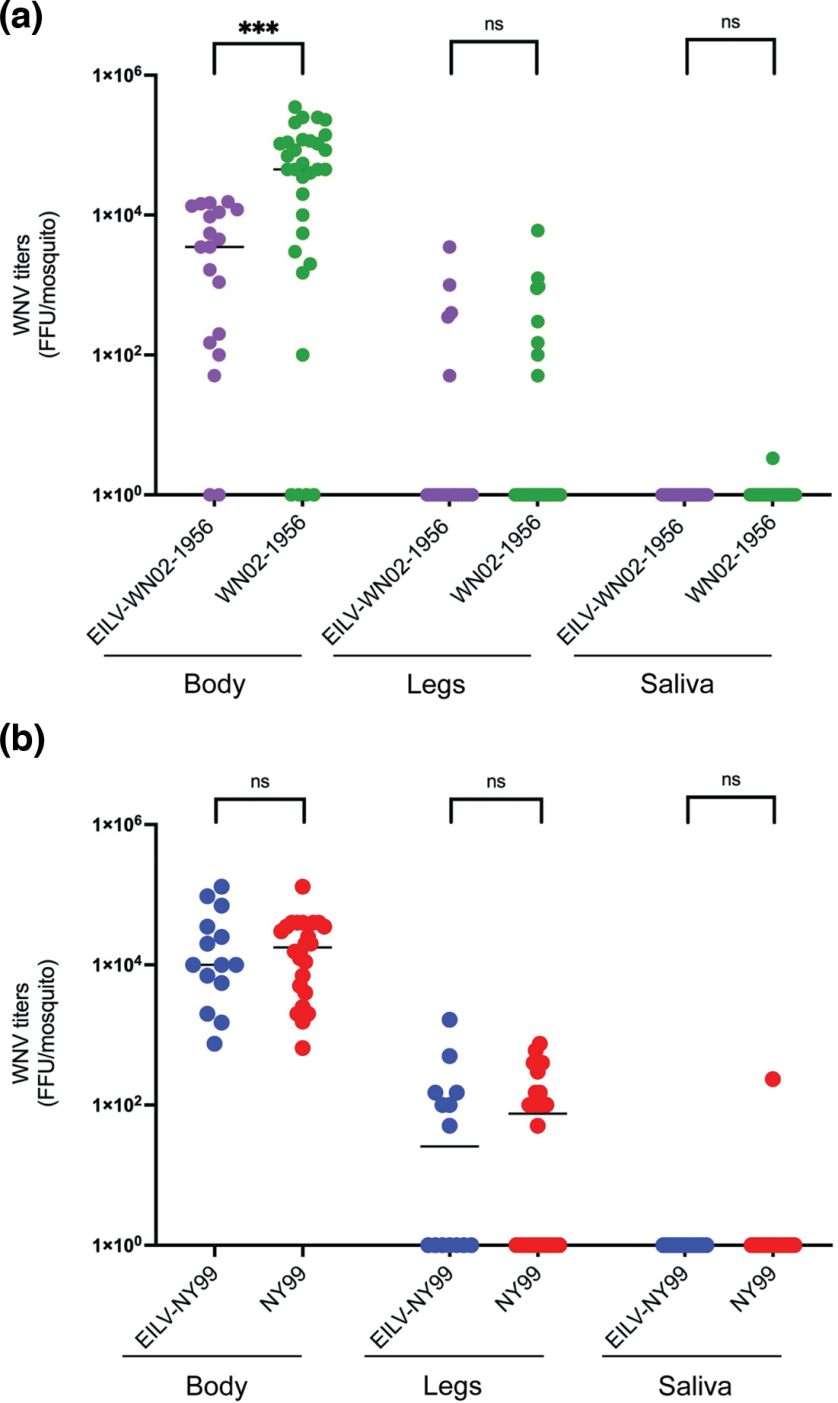
WNV titres of body, leg and saliva samples from EILV-eGFP-infected and mock infected *C. tarsalis* mosquitoes challenged with WN02-1956 or NY99. WNV titres in (**a**) body, (**b**) leg and (**c**) saliva samples from EILV-eGFP-infected (10^7^ f.f.u. ml^–1^) and mock infected *C. tarsalis* mosquitoes challenged with WN02-1956 or NY99 (10^7^ f.f.u. ml^–1^) at 7 days post-superinfection are plotted. Individual points represent a single mosquito sample, while group medians are depicted by horizontal bars. Significance was determined using Mann–Whitney U tests. ***P<0.001.

Moreover, we found that EILV significantly suppressed WN02-1956 body titres in superinfected mosquitoes (Fig. 4, Mann–Whitney U tests, *P*≤0.001). This suppression of WN02-1956 body titres did not result in an overall decrease in WN02-1956 titres in the legs of superinfected mosquitoes (Fig. 4a, *P*>0.05). Similarly, no difference was observed in the NY99 body or leg titres between superinfected and singly infected mosquitoes (Fig. 4b, *P*>0.05).

## Discussion

WNV is a pathogenic arbovirus of public health importance, especially in the USA; however, there are currently no human vaccines or WNV-specific antivirals available against this pathogen [5,12]. The main strategy to decrease the prevalence of this virus is vector control [5,12]. One potential control strategy is the use of ISVs to target pathogenic viruses through SIE [16,22]. The WNV vector *C. tarsalis* is a competent host for the ISV EILV [13]. Therefore, the primary goal of the present study was to determine whether EILV causes SIE against WNV – an unrelated flavivirus – in C6/36 cells and *C. tarsalis* mosquitoes. In the present study, we report that EILV suppresses the titres of both WNV strains WN02-1956 and NY99 in C6/36 cells. In contrast, EILV enhanced the viral infection titres of NY99 in *C. tarsalis* mosquitoes at 3 days post-superinfection but suppressed WN02-1956 infection titres at 7 days post-superinfection. Overall, EILV was found to cause SIE against WNV in both C6/36 cells and *C. tarsalis* mosquitoes but in a strain-specific manner.

Previous studies on the interactions between ISVs and other viruses under superinfection conditions have reported variable results, ranging from interference [17,18, 22, 40,43] and enhancement [39,44] to no effect on the secondary infection [38,45]. We found that the alphavirus EILV caused SIE against the flavivirus WNV in C6/36 cells, irrespective of the m.o.i. of WNV tested in our study. Superinfection of EILV-infected C6/36 cells with either the WNV strain WN02-1956 or NY99 at an m.o.i. of 0.1 or 0.01 suppressed WNV viral titres as early as 48–72 h post-superinfection. Although NY99 titres at an m.o.i. of 0.01 showed signs of recovery towards the final timepoint in our study, these findings are similar to previous findings indicating that ISVs cause heterologous interference *in vitro* against pathogenic viruses belonging to other families [30,35, 46,48]. The ability of EILV to cause SIE is also not limited to WNV; it causes SIE against itself and other alphaviruses, including Sindbis virus, western equine encephalitis virus and CHIKV [22]. This indicates that EILV provides a broad range of protection against secondary infection in cells.

The exact mechanism of SIE remains elusive, but interference at different stages of the life cycle of the secondary virus – including attachment [31,32], penetration [24] and replication [33,34] – by the primary virus can contribute to SIE. We showed that EILV significantly reduced the titre of attached NY99 under superinfection conditions in C6/36 cells. The ability of EILV to interfere with NY99 attachment can be explained by downregulation of WNV receptors or co-receptors after EILV infection in these cells, as previously observed regarding other superinfecting viruses [32,49, 50]. In contrast, the attachment of WN02-1956 was not altered by the presence of EILV in our study. The differences observed between the WNV strains are potentially due to the mutations in the E and NS5 protein coding sequences of the WN02 genotype compared the coding sequences of these proteins in the NY99 genotype [7,8]. These mutations may help superinfecting WN02-1956 to escape or compensate for the interference caused by EILV at the attachment stage in C6/36 cells [9]; however, further studies are needed to confirm the mechanism of strain-specific interference caused by EILV at the attachment stage. Although the interference at the attachment stage by EILV probably contributes to the mechanism of SIE, it is not the main cause because at a later stage in the viral life cycle – internalization – the titres of both superinfecting strains NY99 and WN02-1956 were similar in the presence or absence of EILV.

The RNAi defence pathway is another potential driver of SIE in arthropods [51,52]. However, our use of C6/36 cells excluded small interfering RNA (siRNA), the main antiviral defence pathway, as the root cause of the ability of EILV to suppress WNV titres in these cells because C6/36 cells lack a functional dicer-2 vital for siRNA activation [53]. However, the PIWI-interacting RNA (piRNA) pathway remains active in these cells [53,54]. A local pairwise comparison of the EILV genome with that of WN02-1956 or NY99 confirmed that these viruses do not share sequence similarities of 21–30 nt in length (not shown), necessary for siRNA and piRNA pathways of cross-protection [53,55]. The ability of CHIKV – another alphavirus – to cause heterologous interference was similarly independent of these RNAi pathways [30]. Alternative RNAi pathways, such as microRNAs, tolerate higher sequence mismatches with their targets and can play a role in superinfection exclusion [53]. Small RNA analysis of EILV and WNV superinfection in C6/36 cells may clarify the role of the RNAi pathway in the ability of EILV to suppress WNV infection in these cells.

Many superinfection studies have been performed in cell culture but not in mosquito vectors. In the present study, we found that EILV did not alter the vector competence of either WNV strain in *C. tarsalis* under superinfection conditions at any timepoint in our study. Although EILV did enhance the NY99 whole-body titres in *C. tarsalis,* the WN02-1956 body titres remained unaffected at 3 days post-superinfection. Similarly, a previous study has reported that the ISV Culex flavivirus Izabal enhanced WNV transmission in *C. quinquefasciatus* (Honduras colony) mosquitoes when co-infected [38]. A probable explanation for the enhanced NY99 titres in our study is that NY99 ‘piggybacks’ and infects more cells or cell types with the help of EILV. This strategy is used by the human immunodeficiency virus during co-infection with viruses such as Epstein–Barr virus to expand its tissue tropism in its host [56]. In the present study, at the later timepoint, 7 days post-superinfection, NY99 infection titres were no longer enhanced, whereas WN02-1956 infection titres were suppressed. Previous studies have reported that other ISVs also suppress WNV, but these studies used only one WNV strain to determine SIE [19,38, 40]. The leg and saliva titres of both NY99 and WN02-1956 were not affected by EILV at this timepoint. NY99 and WN02-1956 are genetically distinct, and this genetic variation may explain the different results obtained in the present study [7,9]. The mutations in the WN02 genotype enhance its dissemination in *C. tarsalis* compared with that of NY99 [9]. The decrease in WN02-1956 whole-body titres may be attributed to a need for more shared resources with EILV at a faster rate than NY99 [9]. Further investigation into the effect of individual mutations between NY99 and WN02-1956 or transcriptional differences between NY99 and WN02-1956 under superinfection conditions with EILV may elucidate the strain-specific effect of EILV on WNV in *C. tarsalis*.

A caveat of our study is the limited number of superinfected mosquitoes used in our vector competence assays. *C. tarsalis* infected with EILV appeared less likely to take a second bloodmeal than control mosquitoes. Arbovirus infections can modify the feeding behaviour of their vector, as previously determined with DENV-2-infected *A. aegypti*, which were less likely to refeed than uninfected mosquitoes [43]. Behavioural studies with EILV-infected mosquitoes need to be performed to confirm our observations. Additionally, our EILV–WNV superinfection studies use an acute EILV infection instead of a persistent infection, as observed for most ISVs in nature [16,57, 58]. ISVs are thought to be vertically transmitted from mother to offspring in nature [40,57, 58]; however, although EILV infects the ovaries of *C. tarsalis* following an oral challenge, EILV does not exhibit vertical transmission in laboratory conditions [13]. A persistent EILV infection may result in different outcomes when followed by superinfection with WNV. Alternatively, the intrathoracic route of infection mimics an ISV infection better than the oral route in mosquitoes [13]. However, because EILV infection via the intrathoracic route did not result in the infection of *C. tarsalis* ovaries [13], we used the oral route of infection in the present study to maximize the likelihood of EILV–WNV interaction.

Overall, our study adds to the growing literature on ISVs and their ability to cause SIE against human pathogenic viruses [17,19, 37, 38, 40]. ISVs have been reported to be a potentially safe tool to target pathogenic viruses via SIE in nature [55], but our study demonstrates that although EILV caused heterologous interference against both WNV strains – NY99 and WN02-1956 – in C6/36 cells, SIE in *C. tarsalis* was strain-specific. The WNV strain NY99 no longer circulates in the US population, but its enhancement in *C. tarsalis* by EILV is a dangerous outcome for a potential tool. Additionally, our results demonstrate the importance of testing all circulating virus strains to determine the safety and ability of an ISV to cause SIE. The suppression of WN02-1956 in *C. tarsalis* was modest, with no effect on the dissemination or transmission of the virus, indicating that SIE alone is inadequate as a control strategy. Paratransgenesis [48,59, 60], a strategy by which genetically modified EILV expressing antiviral peptides such as vago [61] is used to target WNV in *C. tarsalis*, may potentially improve the ability of EILV to suppress WNV and increase its specificity.

## supplementary material

10.1099/jgv.0.002017 Fig. S1.

## Abbreviations

BSA: bovine serum albumin
CHIKV: Chikungunya virus
DIR: dissemination rate
dpi: days post-infection
EILV: Eilat virus
FFA: focus-forming assay
IR: infection rate
ISV: insect-specific virus
piRNA: PIWI-interacting RNA
RT: room temperature
RT-qPCR: reverse transcriptase quantitative PCR
SIE: superinfection exclusion
siRNA: small interfering RNA
TE: transmission efficacy
TR: transmission rate
WNV: West Nile virus
