# Supplementary material for: Eilat virus (EILV) causes superinfection exclusion against West Nile virus (WNV) in a strain-specific manner in Culex tarsalis mosquitoes
Source: J Gen Virol. 2024 Aug 27;105(8):002017. doi: 10.1099/jgv.0.002017 (PMC11348563; doi:10.1099/jgv.0.002017)
Supplement: Fig. S1. [file jgv-105-02017-s001.pdf]

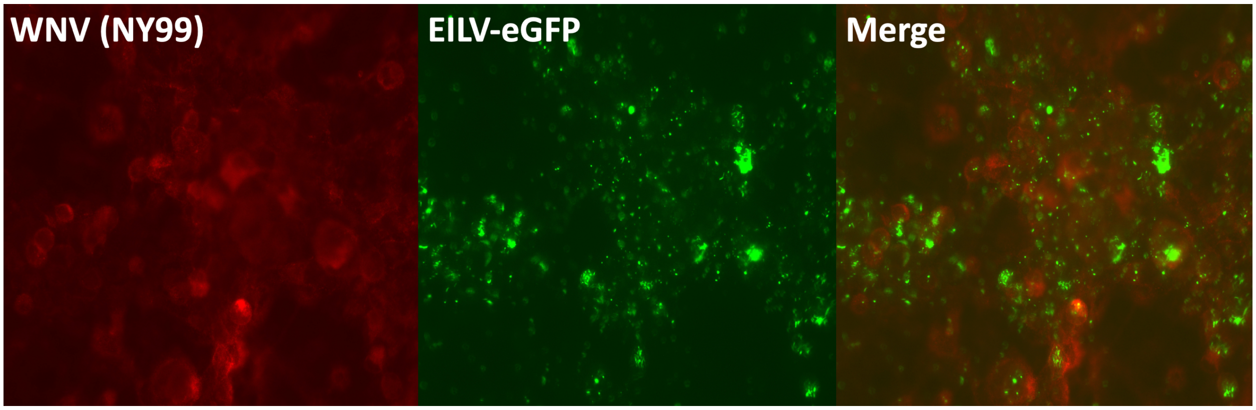

FIG S1. Intracellular co-infection and co-localization of WNV and EILV in mosquito cells.

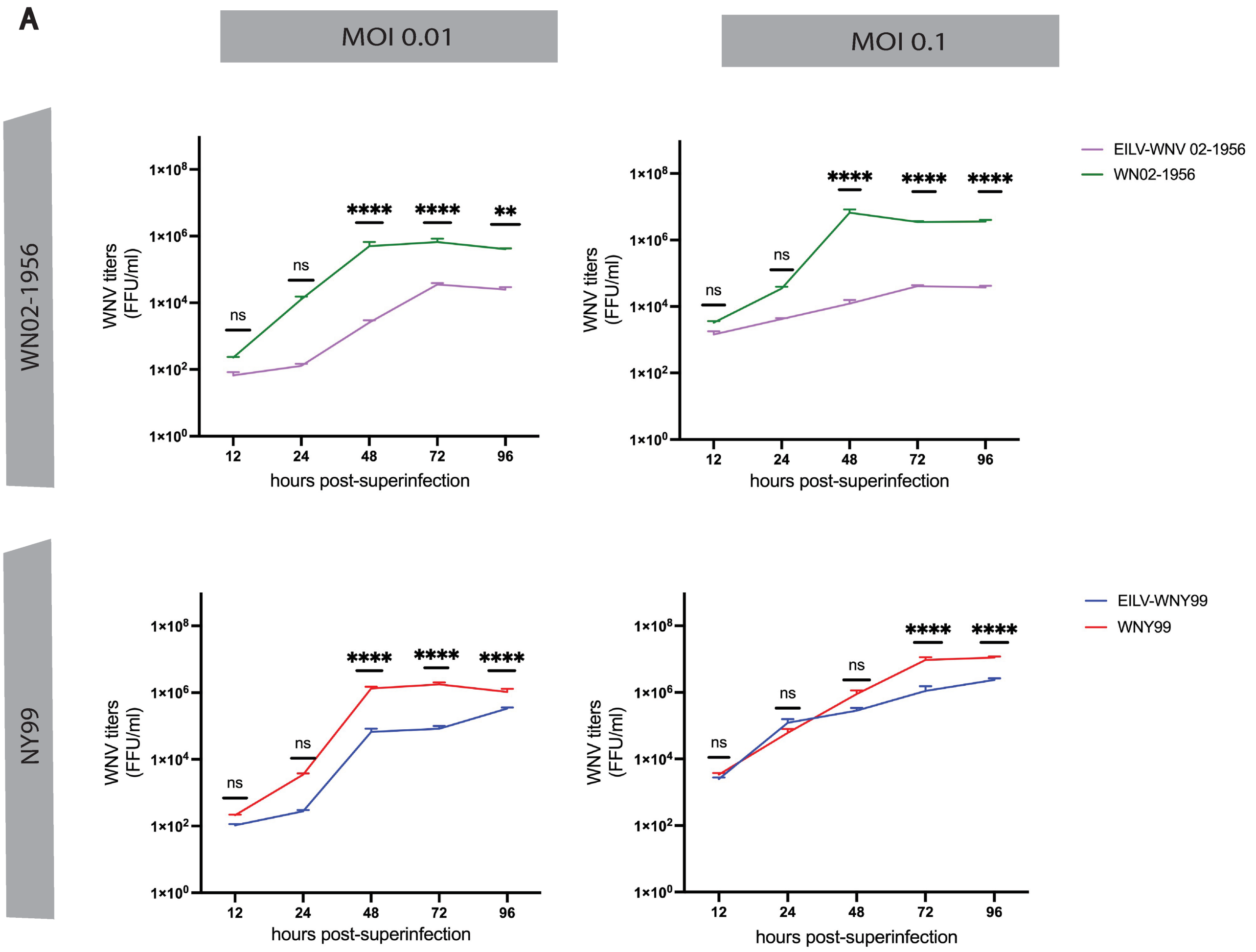

FIG S2 Superinfection exclusion against WNV strains in C6/36 cells by EILV. Titers of WN02-1956 and NY99 at 12, 24, 48,72 and 96 h post-superinfection with EILV was determined by FFA. Each time point reflects the mean of triplicate infection and bar indicate standard deviation at each time point. Statistical significance was evaluated using two-way ANOVA followed by Tukey test. ** *P*<<0.01 and **** *P*<<0.0001.
